# Supplementary material for: Phage homing endonuclease amplifies anti-defense genes to evade bacterial immunity
Source: Nat Commun. 2026 Apr 20;17:3468. doi: 10.1038/s41467-026-71036-4 (PMC13096335; doi:10.1038/s41467-026-71036-4)
Supplement: Supplementary file 1 — Supplementary information [file 41467_2026_71036_MOESM1_ESM.pdf]

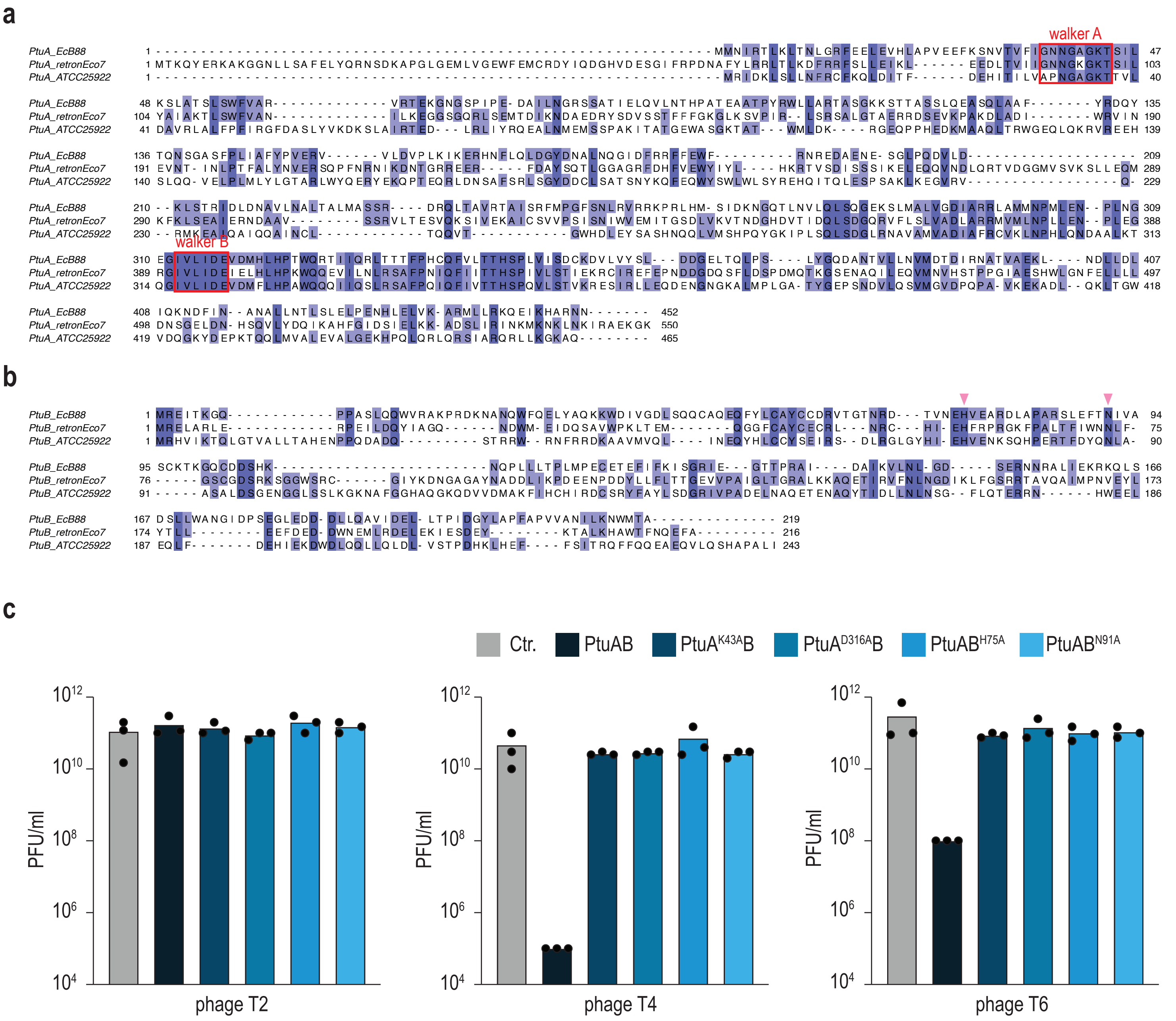

Supplementary Fig. 1 | Antiphage activity of PtuAB mutants from Septu Ec^{B88}.

a and **b**, Multiple sequence alignments of PtuA and PtuB. The sequences of PtuA (**a**) and PtuB (**b**) from the retron Eco7 of *E. coli* ECONIH5, Septu of *E. coli* JBBDAGI-19-0041, and *E. coli* ATCC 25922, were aligned using MAFFT[65] and visualized with JalView[70]. The conserved walker A and walker B motifs are marked in red, while a potential H-N-H motifs in PtuB is highlighted with red triangles.

c, A plaque assay of T-even phages on cells with and without Septu Ec^{B88} from *E. coli* JBBDAGI-19-0041, and with mutations in the predicted active sites of the ATPase or HNH nuclease. Shown is the average of three technical replicates with individual data points overlaid, and no statistical test was performed. Ctr., control. PFU, plaque forming unit.

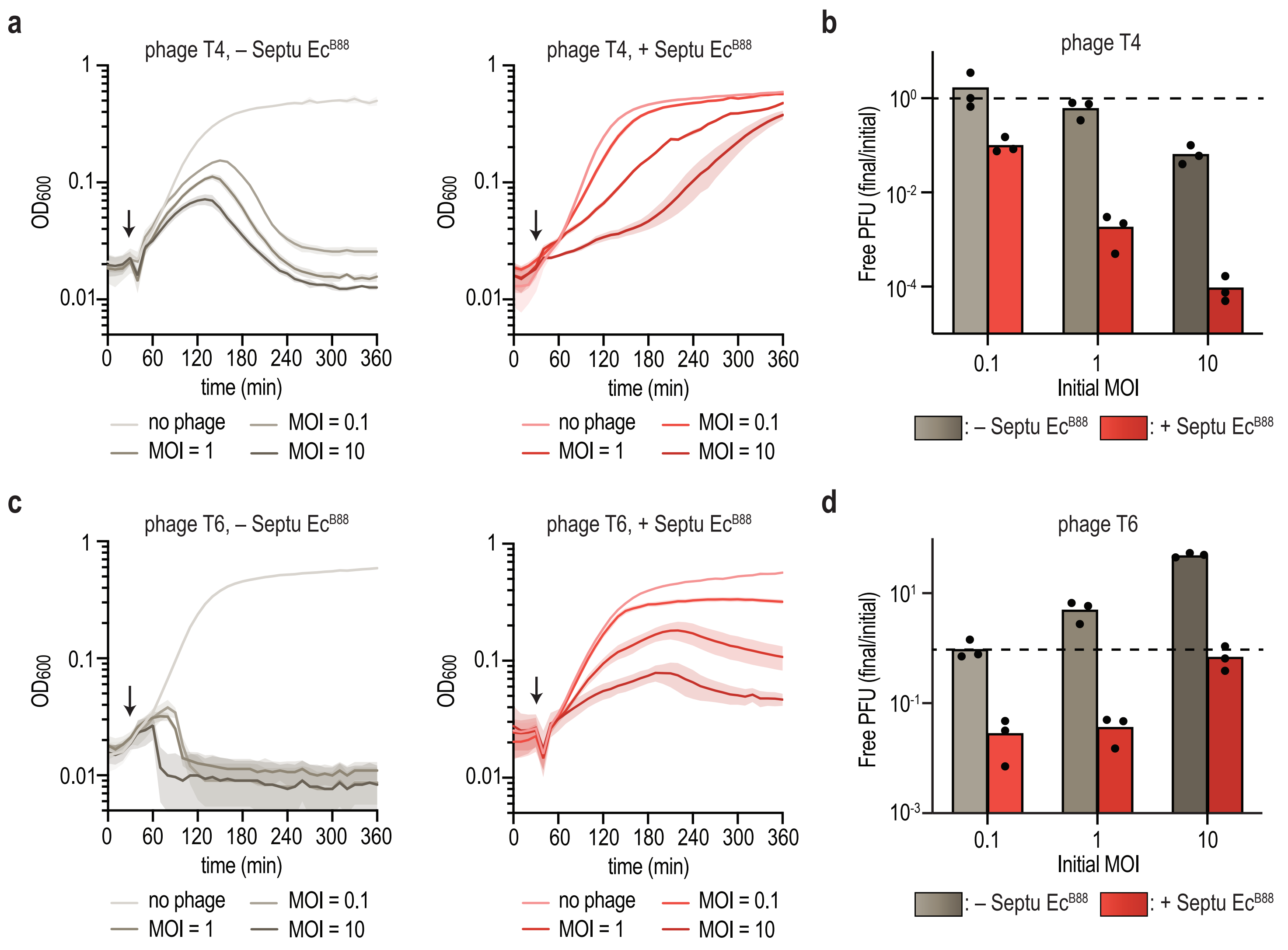

Supplementary Fig. 2 | Liquid phage infection of cells expressing Septu Ec^{B88}.

a and **c**, Growth curves of *E. coli* DH10B expressing an empty vector or Septu Ec^{B88} after infection with either T4 (a) or T6 (c) phage at MOIs of 0.1, 1, or 10. Curves represent the mean of three replicates and shaded areas indicate standard deviation. Arrows indicate the time of phage addition.

b and **d**, Fold change in PFUs of T4 (b) and T6 (b) between pre-infection and 6 h post-infection, measured from the supernatant of *E. coli* DH10B cells expressing either an empty vector or Septu Ec^{B88}. Infections were performed at MOIs of 0.1, 1, or 10. Bars show the mean of three replicates with individual data points overlaid, and no statistical test was performed.

a

SNPs predicted by Snippy

Phage	ESC No.	Locus_tag	Product	Mutation
T6 EcB88 ESC	1			no mutation
	2	KMB99_gp234	tail sheath	V368I
	3	KMB99_gp234	tail sheath	V368I
	4			no mutation

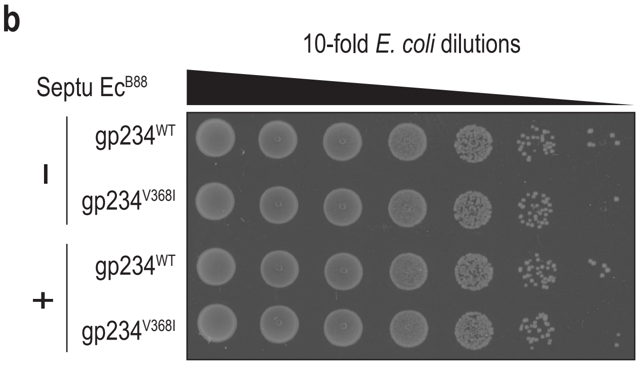

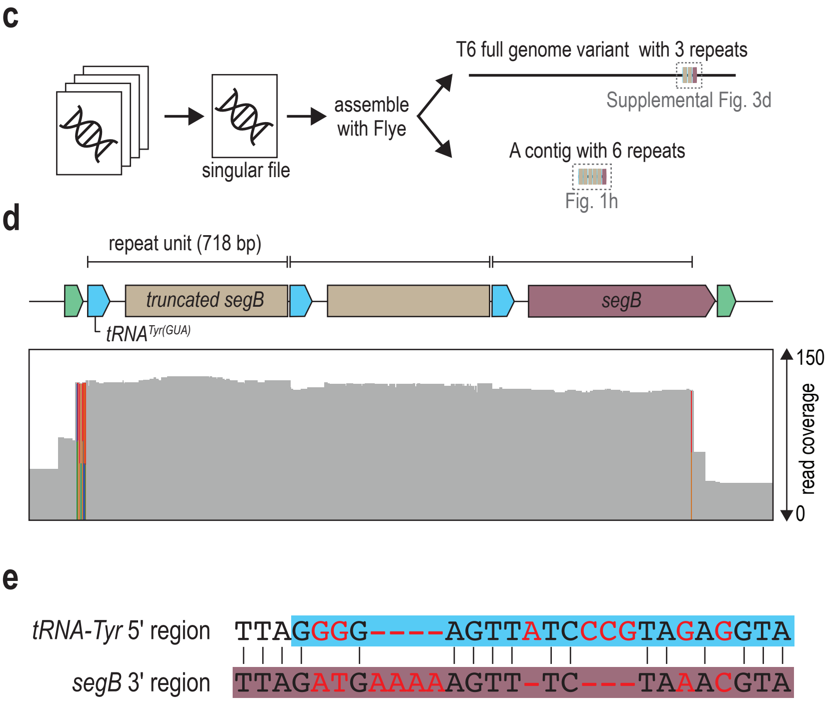

Supplementary Fig. 3 | Mutations occurred in Septu-resistant T6 mutants.

a, SNPs and indels identified in Septu-resistant T6 mutants using Snippy (https://github.com/tseemann/snippy).

b, A toxicity assay on cells co-expressing Septu Ec^{B88} with either the wild-type Gp234 or its mutant (V368I).

c, Diagram illustrating the assembly process for reads obtained from Pac-Bio long-read sequencing.

d, Coverage profile derived from PacBio long-read sequencing for the assembled contig, with the corresponding locus map displayed above. A schematic representation indicates a 718 bp repeat unit, which includes *tRNA*^{Tyr} and a C-terminal truncated version of *segB*.

e, Microhomology observed between the 5' end of *tRNA*^{Tyr} and the 3' end of *segB*, with non-homologous nucleic acids highlighted in red.

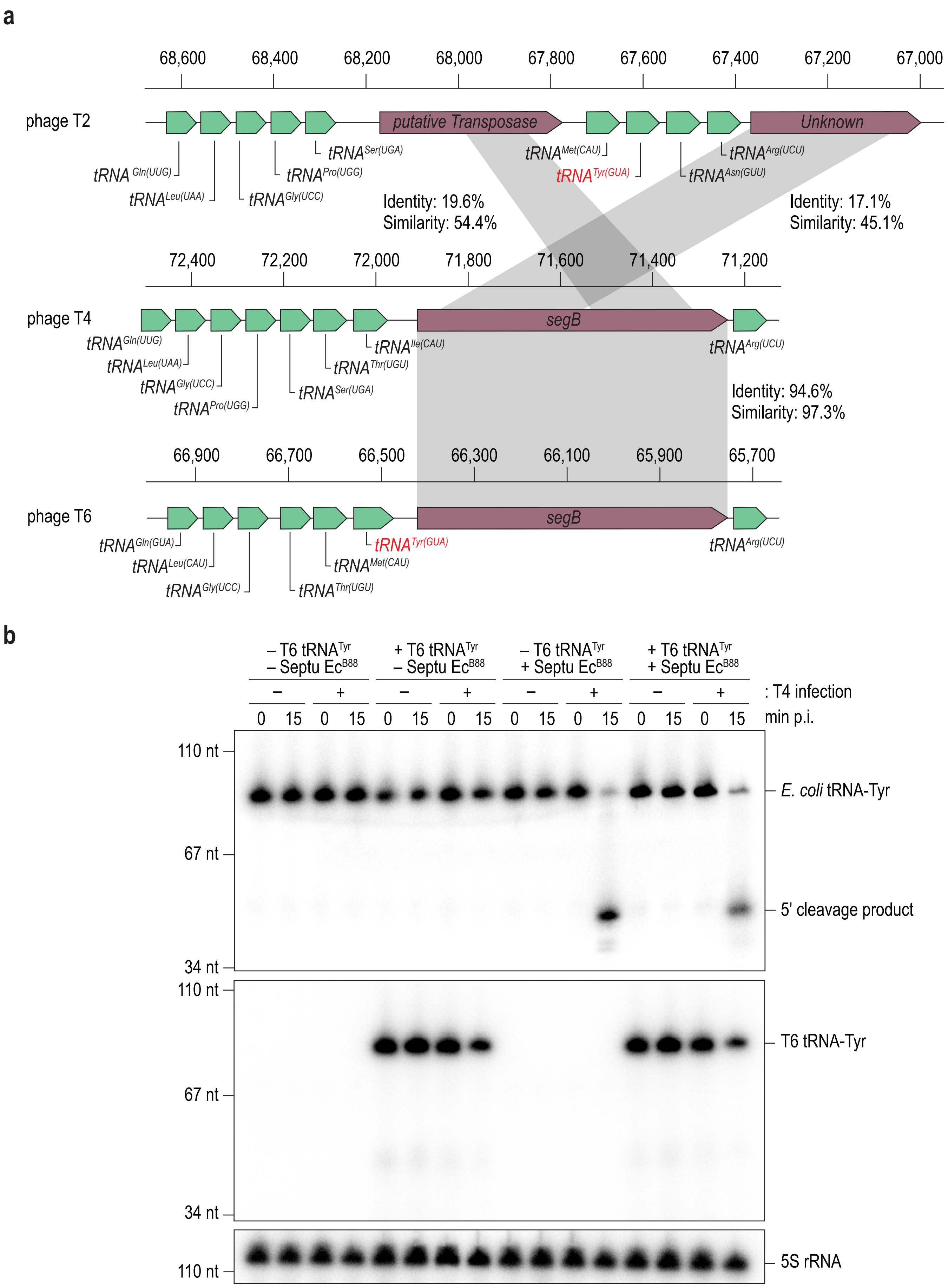

Supplementary Fig. 4 | Genetic organization and expression analysis of $tRNA^{Tyr}$ in T-even phages.

a, The genetic organization of the tRNA array with *segB* in T-even phages. tRNAs and CDSs are shaded in moss green and red purple, respectively. The amino acid sequences of SegB exhibit nearly identical patterns between the T4 and T6 phages, with a 94.6% identity and 97.3% similarity. Phage T2 contains two CDSs, portions of which display weak identities with the SegB amino acid sequence. The $tRNA^{Tyr}$ in T2 and T6 phages are highlighted in red. Genomic coordinates are based on the Enterobacteria phages T2, T4, and T6 complete genomes (GenBank accession no. AP018813.1, AF158101.6, and AP018814.1).

b, Northern blot analysis of the *E. coli* and exogenously expressed T6 phage $tRNA^{Tyr}$ upon T4 phage infection. Total RNAs were resolved by 7M Urea 6% polyacrylamide gel. 5S rRNA is used as a standard. p.i., post infection.

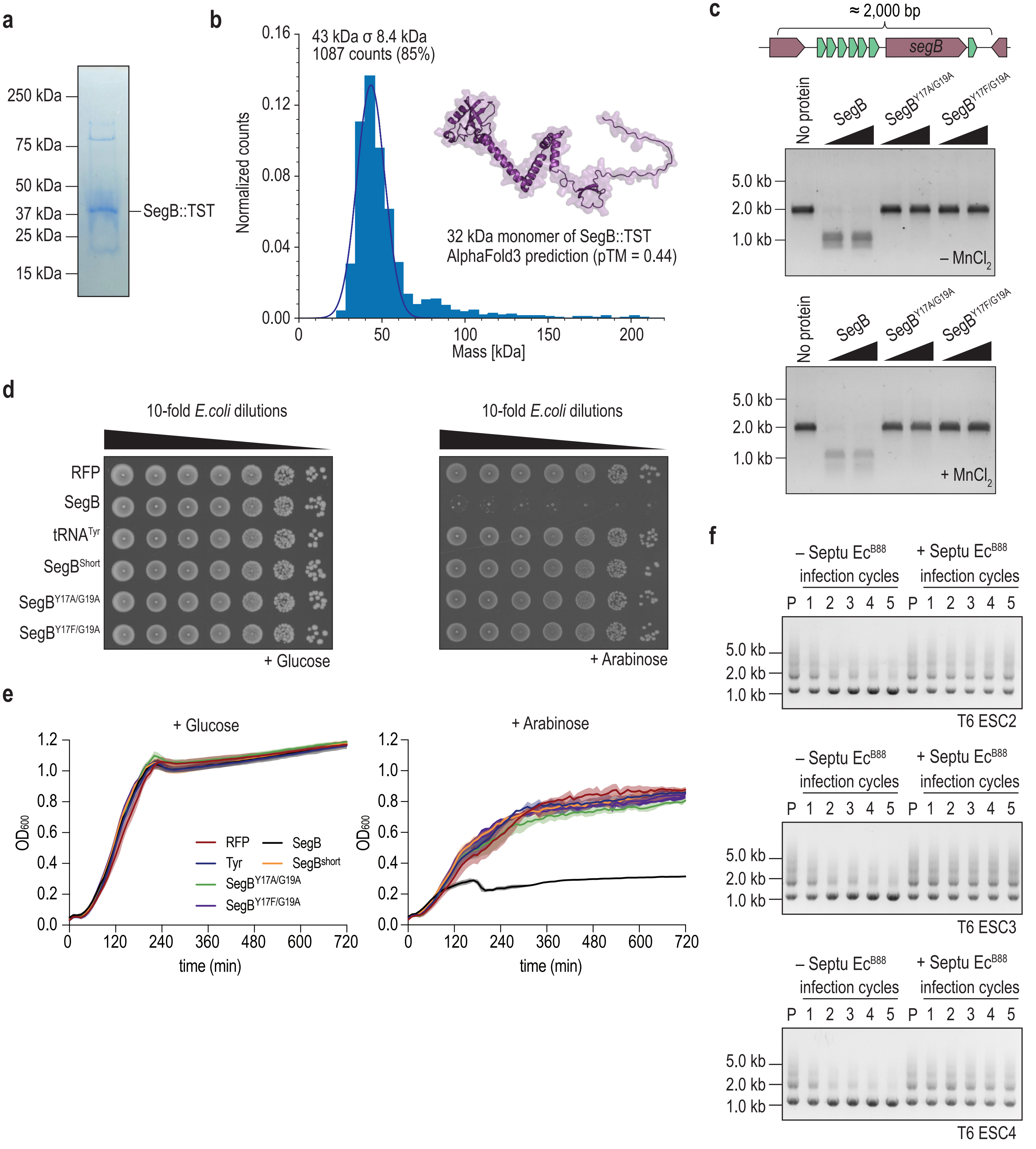

Supplementary Fig. 5 | *in vitro* and *in vivo* analyses of homing endonuclease SegB.

a, Coomassie-stained SDS-PAGE gel displaying purified C-terminally twin-strep-tagged SegB proteins.

b, Mass photometry analysis of purified SegB proteins. The Alphafold3-predicted structure is shown to the right of the graph. TST, twin-strep tag.

c, Nuclease activity assay of SegB and its variants against a PCR product containing the SegB-encoding TRR locus. All reactions contained 1 mM $MgCl_2$, which supports basal SegB activity. $MnCl_2$ was added where indicated to test the reported Mn^{2+} preference of SegB. The PCR fragment used in the assay is indicated in the locus map above.

d, A toxicity assay on cells expressing either RFP, $tRNA^{Tyr}$, SegB, $SegB^{Short}$, $SegB^{Y17A/G19A}$, or $SegB^{Y17F/G19A}$ with either arabinose or glucose.

e, Growth curves of strains expressing either RFP, $tRNA^{Tyr}$, SegB, $SegB^{Short}$, $SegB^{Y17A/G19A}$, or $SegB^{Y17F/G19A}$ with or without arabinose induction. Shaded regions indicate the standard deviation across independent technical replicates (n = 3).

f, Results of gel electrophoresis for PCR products obtained from the lysate of Septu Ec^{B88} escaper mutants ESC2, ESC3, and ESC4 in the experimental evolution via serial passaging.

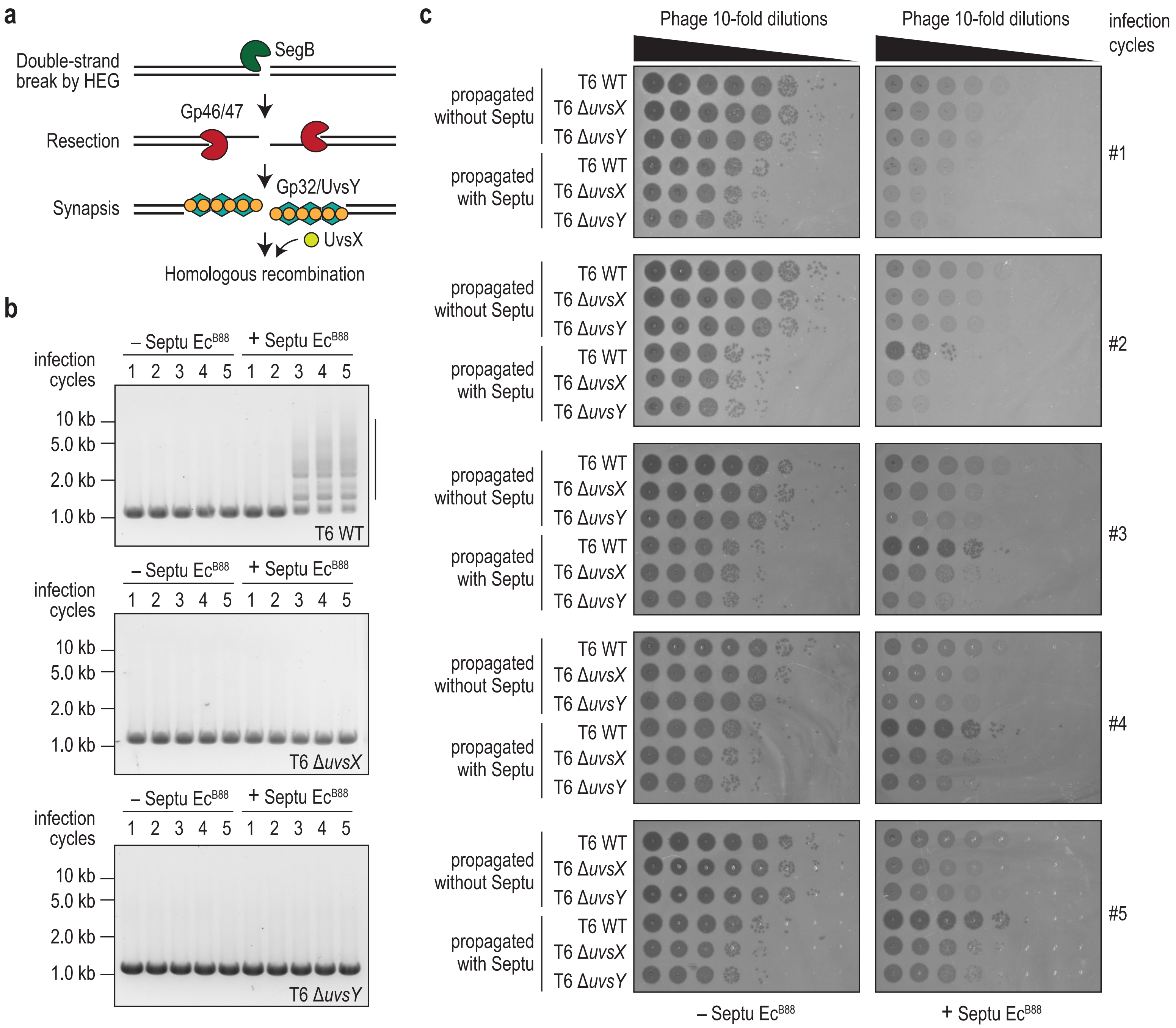

Supplementary Fig. 6 | Phage homologous recombination system is essential for segmental amplification of tRNA^{Tyr}.

a, A schematic representation of the presynapsis pathway in phage homologous recombination. Gp46/47 resects dsDNA ends to release a 3' ssDNA tail. The exposed ssDNA tail is sequestered by the ssDNA-binding protein Gp32 and the recombination mediator protein UvsY to form a tripartite complex. This complex recruits RecA-like protein UvsX; homologous dsDNA molecules invade the presynaptic filament.

b, Results of gel electrophoresis for PCR products obtained from the lysate of T6 wild type, Δ*uvsX*, or Δ*uvsY* mutants in the experimental evolution via serial passaging. Segmental amplification of the relevant locus was highlighted by line.

c, Evaluation of infection efficiency for T6 wild type, Δ*uvsX*, and Δ*uvsY* mutants with plaque assays. Tenfold serial dilutions of phages from the supernatant of the serial passaging experiments were applied onto a lawn of *E. coli* DH10B, either harboring an empty vector or Septu Ec^{B88}.

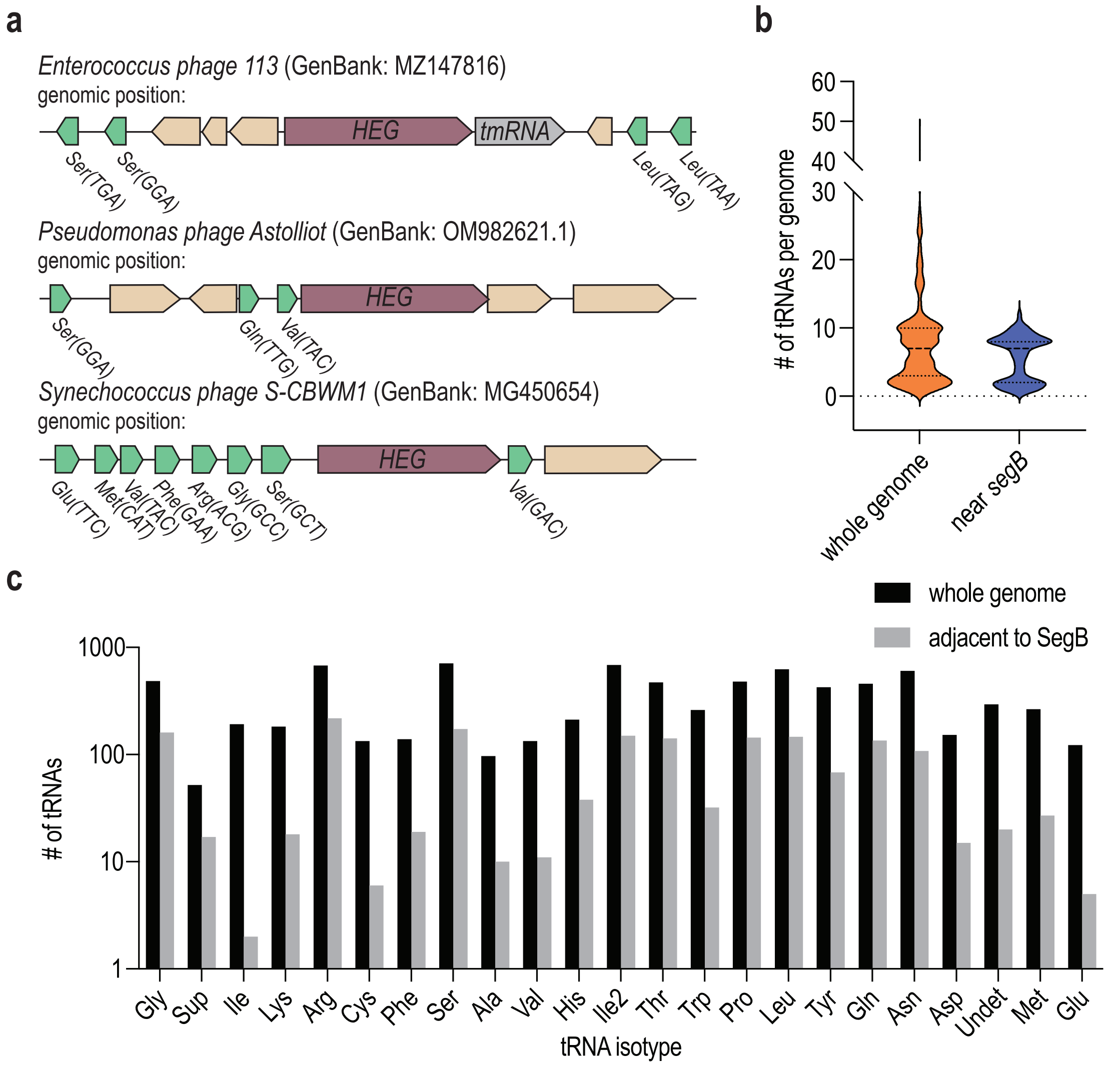

Supplementary Fig. 7 | Co-occurrence of *segB* and tRNAs in various phage genomes.
a, Additional examples of SegB and tRNA colocalization. The names of the phages and the accession numbers of the relevant genomes in the GenBank database are indicated at the top. HEG, homing endonuclease gene.
b, Violin plot depicting the number of tRNAs per genome across entire genomic regions or within 1000 bp upstream or downstream of *segB* genes.
c, The total count of distinct tRNA isotypes found across entire genomic regions or within 1000 bp upstream or downstream of *segB* genes in 1,832 phages containing SegB homologs.

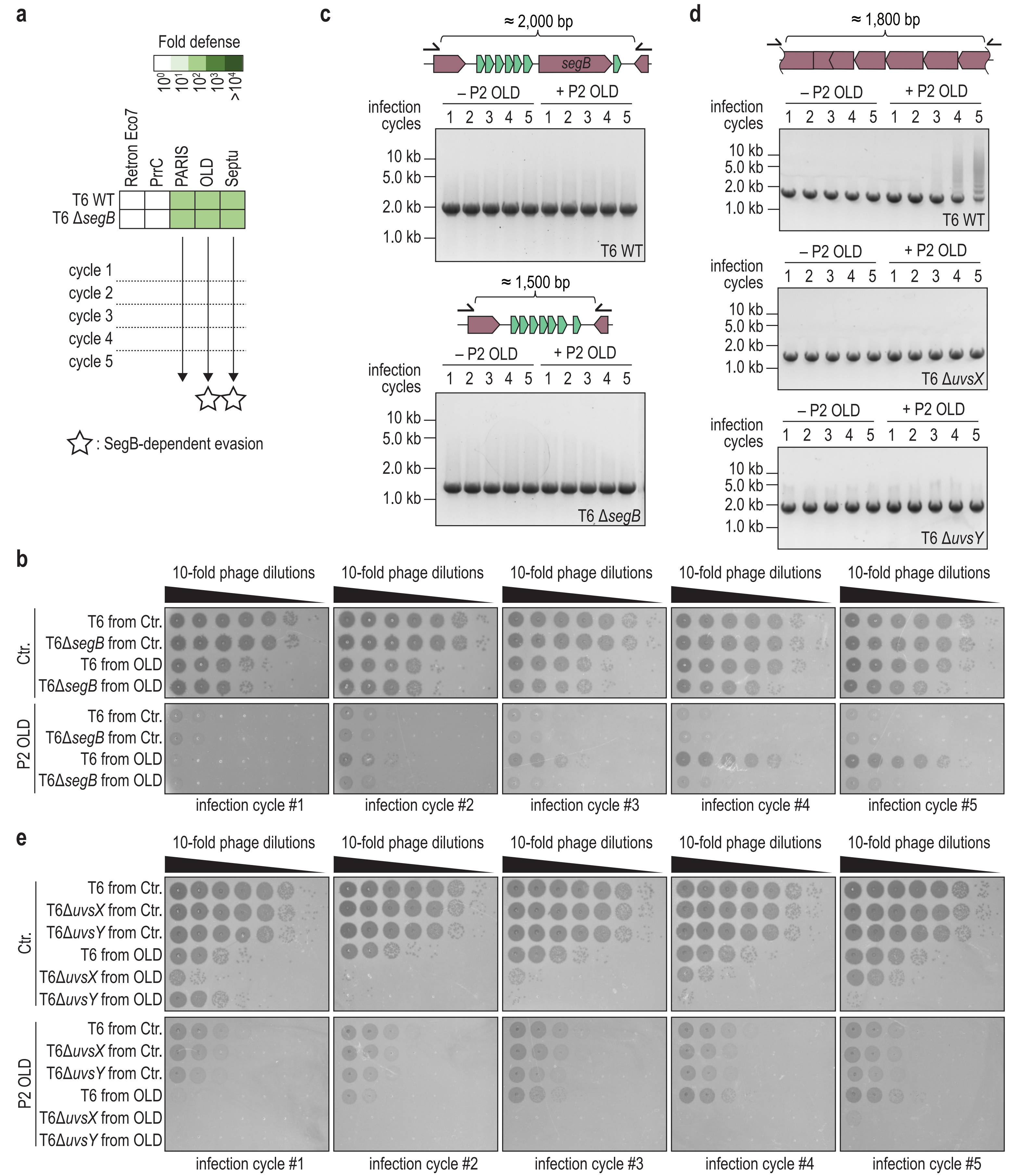

Supplementary Fig. 8 | SegB-dependent evasion of OLD immunity.

a, Heatmap illustrating the defense profiles of tRNA-targeting defense systems against T6 wild-type and its Δ*segB* derivatives. A schematic diagram showing the experimental evolution of serial passaging of T6 phage is shown. Arrows denote infection cycles. Stars indicate SegB-dependent evasion of defense systems. Cycles 1–5 correspond to the first through fifth rounds of the experimental evolution, in which fresh *E. coli* cultures were sequentially infected with supernatants derived from the previous cycle.

b, Evaluation of infection efficiency for T6 wild type and its Δ*segB* derivative using plaque assays against the OLD defense system. Tenfold serial dilutions of phages from the supernatant of the serial passaging experiments were applied onto a lawn of *E. coli* DH10B, either harboring an empty vector or OLD.

c, Gel electrophoresis results for the PCR products obtained from T6 phage wild type or Δ*segB* lysates in the experimental evolution via serial passaging. Primer pairs are displayed in the corresponding locus map above.

d, Evaluation of infection efficiency for T6 wild type, Δ*uvsX*, and Δ*uvsY* mutants with plaque assays. Tenfold serial dilutions of phages from the supernatant of the serial passaging experiments were applied onto a lawn of *E. coli* DH10B, either harboring an empty vector or OLD.

e, Results of gel electrophoresis for PCR products obtained from the lysate of T6 wild type, Δ*uvsX*, or Δ*uvsY* mutants in the serial passaging experiments.

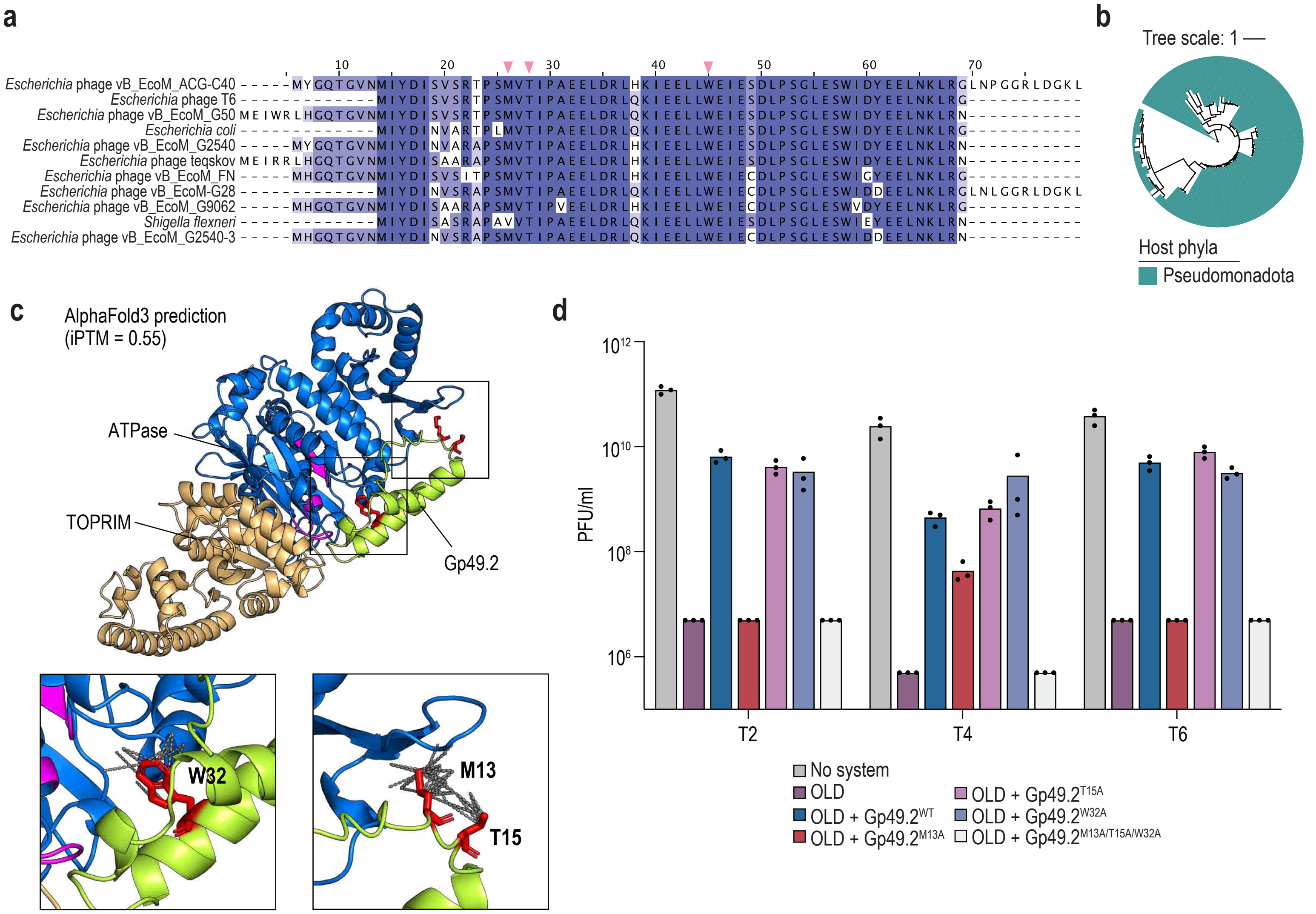

Supplementary Fig. 9 | Functional analysis of the OLD inhibitor Gp49.2.

a, Multiple sequence alignment of Gp49.2 homologs identified by BLASTp. Sequences were filtered using a sequence identity cutoff of 0.9. Red triangles indicate residues targeted for mutagenesis (M13, T15, and W32). Sequences were aligned using MAFFT[65] and visualized with JalView[70].

b, Phylogeny and distribution of Gp49.2 homologs.

c, Interaction between P2 OLD and Gp49.2 as predicted by AlphaFold3 (ipTM = 0.55)[34]. The ATPase and TOPRIM domains of OLD are shown in marine and yellow, respectively. Walker A and Walker B motifs are highlighted in magenta. Insets show close-up views of the predicted interaction surface and the positions of residues M13, T15, and W32 in Gp49.2.

d, Plaque-forming units of T2, T4, and T6 phages on cells co-expressing OLD with an RFP, wild-type Gp49.2, or the indicated Gp49.2 mutants. Bars represent the mean of three technical replicates with individual data points overlaid, and no statistical test was performed.

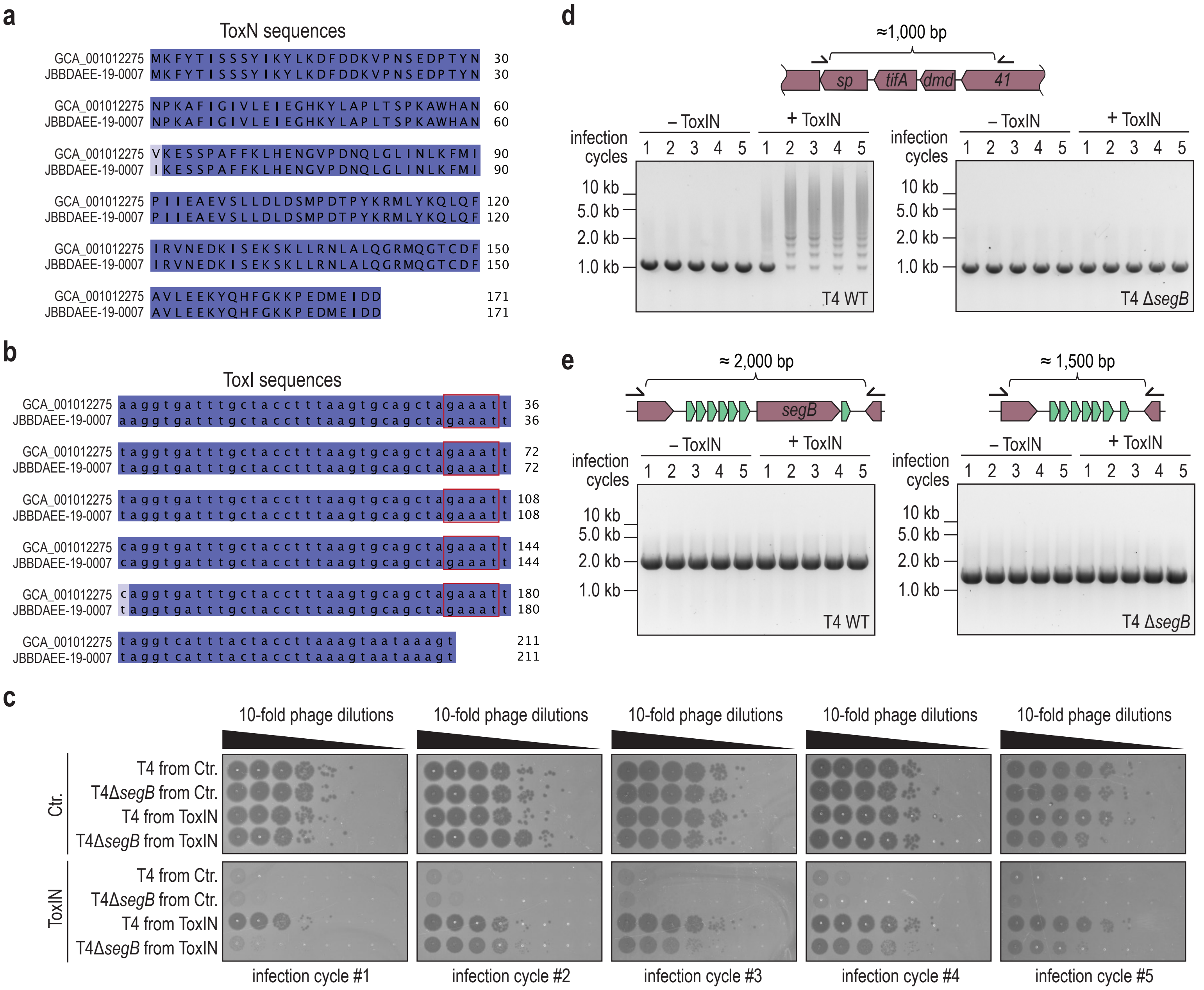

Supplementary Fig. 10 | SegB-dependent evasion of ToxIN immunity in phage T4.

a, Sequence alignment of ToxN sequences from *E. coli* GCA_001012275 and JBBDAEE-19-0007.

b, Sequence alignment of toxI sequences from *E. coli* GCA_001012275 and JBBDAEE-19-0007. The ToxN cleavage motifs were marked in red. Sequences were aligned using MAFFT[65] and visualized with JalView[70].

c, Evaluation of infection efficiency for T4 wild type and its Δ*segB* derivative using plaque assays against the ToxIN defense system. Tenfold serial dilutions of T4 phages from the supernatant of the serial passaging experiments were applied onto a lawn of *E. coli* DH10B, either harboring an empty vector or ToxIN.

d and **e**, Results of gel electrophoresis for PCR products obtained from the lysate of T4 wild type or its Δ*segB* mutants in the experimental evolution via serial passaging.
