# Supplementary material for: Phage homing endonuclease amplifies anti-defense genes to evade bacterial immunity
Source: Nat Commun. 2026 Apr 20;17:3468. doi: 10.1038/s41467-026-71036-4 (PMC13096335; doi:10.1038/s41467-026-71036-4)
Supplement: Supplementary file 2 — Description of Additional Supplementary Files [file 41467_2026_71036_MOESM2_ESM.pdf]

Description of Additional Supplementary Files

Supplementary Data 1 | SITE-seq 5′ differential peaks

Supplementary Data 2 | Homologs of the T6 phage SegB identified by HMM-based search across viral proteomes.

Supplementary Data 3 | tRNA genes encoded in phage genomes containing SegB homologs.

Supplementary Data 4 | Homologs of the T6 phage Gp49.2 identified by HMM-based search across viral proteomes.

Supplementary Data 5 | Phages used in this study.

Supplementary Data 6 | Plasmids used in this study.

Supplementary Data 7 | Oligonucleotides used in this study.

Supplementary Data 8 | Sequence of the cloned defense systems (Septu Ec^{B88}, ToxIN, OLD, PARIS)
